# Unravelling Structure, Localization, and Genetic Crosstalk of KLF3 in Human Breast Cancer

**DOI:** 10.1155/2020/1354381

**Published:** 2020-12-28

**Authors:** Khushbukhat Khan, Sadia Safi, Asma Abbas, Yasmin Badshah, Erum Dilshad, Mehak Rafiq, Kainat Zahra, Maria Shabbir

**Affiliations:** ^1^Department of Healthcare Biotechnology, Atta-ur-Rahman School of Applied Biosciences, National University of Sciences and Technology, Islamabad 44000, Pakistan; ^2^Department of Biological Sciences, Capital University of Science & Technology, Islamabad 44000, Pakistan; ^3^Research Centre for Modelling & Simulation (RCMS), National University of Sciences and Technology, Islamabad 44000, Pakistan

## Abstract

Breast cancer is the most prevailing disease among women. It actually develops from breast tissue and has heterogeneous and complex nature that constitutes multiple tumor quiddities. These features are associated with different histological forms, distinctive biological characteristics, and clinical patterns. The predisposition of breast cancer has been attributed to a number of genetic factors, associated with the worst outcomes. Unfortunately, their behavior with relevance to clinical significance remained poorly understood. So, there is a need to further explore the nature of the disease at the transcriptome level. The focus of this study was to explore the influence of Krüppel-like factor 3 (KLF3), tumor protein D52 (TPD52), microRNA 124 (miR-124), and protein kinase C epsilon (PKC*ε*) expression on breast cancer. Moreover, this study was also aimed at predicting the tertiary structure of KLF3 protein. Expression of genes was analyzed through real-time PCR using the delta cycle threshold method, and statistical significance was calculated by two-way ANOVA in Graphpad Prism. For the construction of a 3D model, various bioinformatics software programs, Swiss Model and UCSF Chimera, were employed. The expression of KLF3, miR-124, and PKC*ε* genes was decreased (fold change: 0.076443, 0.06969, and 0.011597, respectively). However, there was 2-fold increased expression of TPD52 with *p* value < 0.001 relative to control. Tertiary structure of KLF3 exhibited 80.72% structure conservation with its template KLF4 and was 95.06% structurally favored by a Ramachandran plot. These genes might be predictors of stage, metastasis, receptor, and treatment status and used as new biomarkers for breast cancer diagnosis. However, extensive investigations at the tissue level and in *in vivo* are required to further strengthen their role as a potential biomarker for prognosis of breast cancer.

## 1. Introduction

Breast cancer is a malignancy that originates from breast tissue. It comprises a mass of cells that along with invading surrounding tissues of the body also metastasize to distant parts such as the lungs, bone marrow, regional lymph nodes, and liver [1]. It is presumed that breast cancer results from the accumulation of several genetic aberrations, resulting in inactivation of tumor suppressor genes and activation of protooncogenes. Current evidences from literature indicate that approximately 10-20% of cases are due to a germline mutation in a gene that leads to increased predisposition for breast cancer [2, 3] while 15-20% of breast cancer incidences are familial [4]. Other than germ line genes, dysregulation in several other genes encoding tumor suppressor proteins, transcription regulators, microRNAs, kinases, and phosphatases has led to breast cancer [5, 6].

Genome-wide studies of human cancer have shown that Receptor Tyrosine Kinase (RTK) and G Protein-Coupled Receptor (GPCR) are the major receptors that regulate the effector protein [7, 8]. Phosphoinositide 3-kinases (PI3K) and Kras via channeling signal transduction from various growth factors and cytokines into intracellular signaling lead to the major activation of Akt and downstream pathways that will ultimately result in cancer [9]. Through a literature review, it is also revealed that KLF3, TPD52, PKC*ε*, and miR-124 interact with different downstream signaling molecules of these pathways in different cancers and lead to cancer proliferation, stage progression and metastasis, and drug resistance [10–15].

KLF3 is a DNA-binding transcription repressor that belongs to the seventeen-member family of KLFs. It contains three C_2_H_2_ zinc finger elements at the C-terminal region. It binds to GC-rich elements and CACCC-binding motif at specific DNA through these motifs and regulates erythropoiesis, lymphopoiesis (particularly B lymphocyte), and adipogenesis [16, 17]. KLF3 engages with corepressor C-terminal binding protein (CtBP) and recruits other repressive cofactors, like histone deacetylases (HDAC), histone methyltransferases (HMT), and histone lysine-specific demethylases (HLSD) [18]. KLF3 acts as an oncogene in cervical and lung tumors [11] while in acute myeloid leukemia and pancreatic and prostate cancer, it displays tumor-suppressive behavior [19].

miR-124 is one of the widely expressed microRNAs in the nervous system, which is more abundant in differentiating neurons of the brain, spinal cord, and retina and lasts in mature neurons [20]. Previous reports had highlighted its role as oncomiRs and oncosuppressors in various cancers. Its dysregulation is associated with aberrant signal transduction in various cellular pathways in cancers such as STAT3 in glioblastoma [21], phosphoinositide 3-kinase (PI3K) and protein kinase B (PKB) (also known as Akt) in prostate cancer, and Notch signaling pathway in gastric cancer [22]. TPD52 is a 180 amino acid-long, highly charged acidic protein. Its genetic sequence is located on chromosome 8 (8q21). It is actually a peripheral membrane and cytosolic protein of the mammalian gene family that performs several biological functions including vesicle trafficking and lipid metabolism [23]. Its aberrant expression in several cancers such as prostate, ovarian, and colorectal cancer, adenocarcinoma, hepatocellular carcinoma, and renal and adrenocortical carcinoma had been widely reported [12].

PKC*ε* is one of the isoforms of PKC which is the family of serine/threonine kinases [24]. The gene of PKC*ε* (PRKCE) resides on chromosome 2 (2p21). It needs diacylglycerol/phobol esters and Ca^2+^ for its activity [25]. PKC*ε* is involved actively in adhesion, migration, transportation, proliferation, differentiation, gene expression, inflammation, immune regulation, secretory processes, and various signal transduction processes [26]. PKC*ε* involvement in the onset of various tumors such glioblastoma; renal, prostate, colorectal, and follicular thyroid cancer; and non-small-cell lung carcinoma is also reported [27]. To date, no study is conducted that investigated the coexpression profile of TPD52, KLF3, miR-124, and PKC*ε*. So, the goal of the current study is to analyze the expression pattern of TPD52, KLF3, miR-124, and PKC*ε* in breast cancer patients as well as to predict their possible correlation with clinicopathological characteristics of breast cancer. Furthermore, the three-dimensional structure of KLF3 is not predicted yet. In this study, we have also predicted the tertiary structure of KLF3 by Swiss modeling. Understanding the expression profile of these genes will provide further insight into complex molecular dynamics. Additionally, this data can be a step towards constructing a molecular diagnostic strategy for breast cancer.

## 2. Materials and Methods

### 2.1. In Vitro Method

#### 2.1.1. Blood Sample Collection

After approval from the Institutional Review Board of Atta-ur-Rahman School of Applied Biosciences (Ref. no: IRB-110) and Combined Military Hospital, blood samples were collected from 50 breast cancer patients as well as 50 controls. All the procedures were performed according to the guidelines provided by ethical review board. A consent form was signed by patients who were willing to give their blood.

History forms of patients were filled after collecting all the relevant information which comprised patient's age, tumor grade, cancer type, treatment status (pretreated and naïve-treated), and receptor subtypes. Other than pathological features, information regarding family history, breastfeeding, post/premenopausal age, and smoking/alcohol consumption was also collected from patients.

#### 2.1.2. RNA Isolation and Synthesis of cDNA

RNA was extracted from 50 collected blood samples of breast cancer patients as well as 50 controls, i.e., healthy individuals, using the TRIzol reagent manufactured by Thermo Fisher Scientific (Waltham, MA, USA). cDNA was synthesized from the extracted mRNA using MLV reverse transcriptase (Promega). The synthesized cDNA was stored at -20°C.

#### 2.1.3. Real-Time PCR

Expression analysis was performed by a real-time PCR (qPCR) quantification assay (Applied Biosystems 7300) using SYBR Premix ExTaq II (TaKaRa). The relative expression of TPD52, KLF3, miR-124, and PKC*ε* was calculated using the Livak method (2 − ΔΔCT) while the beta-actin gene was used as the housekeeping gene.

Primers used for understudied genes are as follows: KLF3 forward 5′-TGTCTCAGTGTCATACCCATCT-3′ and reverse 5′-CCTTCTGGGGTCTGAAAGAACTT-3′, miR-124 forward 5′-GATACTCATAAGGCACGCGG-3′ and reverse 5′-GTGCAGGGTCCGAGGT-3′, TPD52 forward 5′-GAGGAAGGAGAAGATGTTGC-3′ and reverse 5′-GCCGAATTCAAGACTTCTCC-3′, and PKC*ε* forward 5′-AGCCTCGTTCACGGTTCT-3′ and reverse 5′-TGTCCAGCCATCATCTCG-3′.

#### 2.1.4. Statistical Analysis

One-way and two-way ANOVA was performed via Graphpad Prism 6.0 for analysis of association between relative expression of TPD52, KLF3, miR-124, and PKC*ε* and clinicopathological characteristics of breast cancer.

#### 2.1.5. In Silico Method

The 345-amino acid sequence of KLF3 (Homo sapiens) in the FASTA format was retrieved from the National Center for Biotechnology Information (NCBI) having reference sequence id NP_057615.3. The multiple sequence alignment tool “Clustal Omega” was used for aligning homologous primary protein sequences of KLF family members by progressive alignments [28]. The alignment revealed conserved domains within the family. Various different tools were used to predict secondary structure such as Porter 5.0 [29], SOPMA [30], and I-TASSER [31]. For better understanding of evolutionary conservation, phylogenetic analysis of whole KLF family proteins was performed by using Molecular Evolutionary Genetics Analysis version 7.0 (MEGA7) software [32]. For the construction of 3D protein structure, KLF4 was selected as a template (the only predicted structure of the KLF family). The target sequence (KLF3) in the FASTA format and the PDB file of template (KLF4) were added and run in Swiss Model. The obtained 3D structure was visualized in UCSF Chimera 1.13 using template structure as the reference.

## 3. Results

### 3.1. Clinicopathological Features of Breast Cancer and Patient Distribution

After written and oral contest, blood samples of 50 breast cancer patients were collected. Patients were categorized according to clinical features of the diseases and risk factors (see Tables 1 and 2). Clinical features of the disease included receptor status, cancer stage and type, and treatment status while risk factors included were family history, age, breast feeding status, pre- or postmenopause, and smoking or alcohol consumption habits.

### 3.2. Subcellular Localization of KLF3

**Figure 1:**
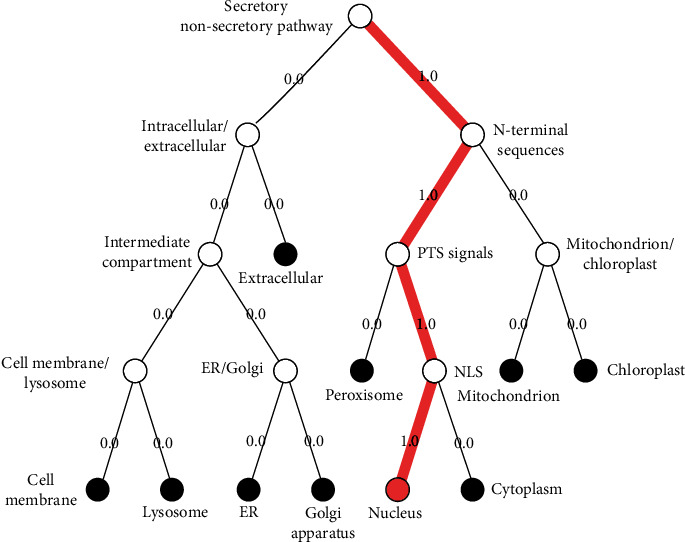
Pathway that follows subcellular localization of KLF3 protein by DeepLoc-1.0. Various locations are shown, and each follows different pathways and signals. However, KLF3 protein is located in the nucleus and is directed towards the nucleus by committing PTS signals and NLS signals (depicted by 1.0 score).

The subcellular localization of the KLF3 (NP_057615.3) protein is within “nucleus” (see Figure 1) as predicted by the server DeepLoc-1.0 that covers various organelles and also differentiates between them for localizations. The location of KLF3 is then checked and confirmed through the UniProtKB database.

### 3.3. Conserved Domains

**Figure 2:**
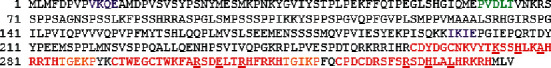
The amino acid sequence of human KLF3. The PVDLT (highlighted in green) region that is a binding site for CtBP, sumoylation motifs (highlighted in purple), zinc finger linker motifs (shown in orange), and amino acid residues (underlined in red) within zinc fingers (depicted in red) that facilitate in making contact with DNA are present in the primary structure of KLF3.

The result summary from Clustal Omega has revealed that within the amino acid sequence of KLF3 protein, the areas that link three zinc fingers are zinc linker motifs with sequence: TGEKP and TGIKP. The amino acids at position 61-65 make the CtBP-binding motif while the motifs PVKQE and IKIE are involved in repression activity of KLF3 (see Figure 2).

**Figure 3:**
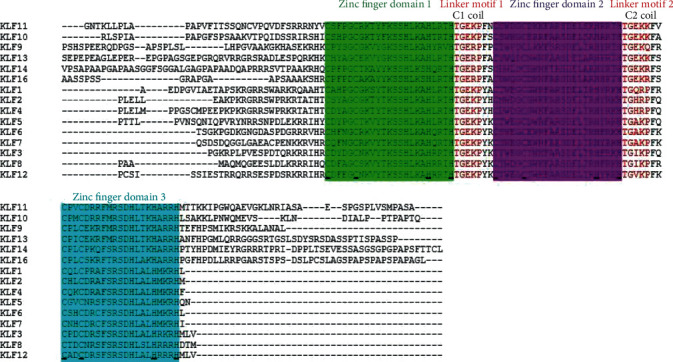
Sequence alignment of Krüppel-like factors depicting conserved domains obtained from Clustal Omega. Certain sequence alignments have to be deleted during formatting. Zinc figure domain 1 (labeled as green), zinc figure domain 2 (labeled as purple), and zinc figure domain 3 (labeled as blue) have been highlighted with underlined C_2_H_2_ elements that coordinate with zinc ions. Different residues of zinc fingers are involved in making coils predicted by I-TASSER, Porter 5.0, and SOPMA. In between the three zinc fingers, two linker motifs are present for link fingers (highlighted in red).

Among all members of KLFs exist three regions that correspond to zinc finger domains with C_2_H_2_-type elements showing coordination with three zinc ions (see Figure 3). These all domains have regulatory function via interacting with promoters of targeted genes. The presence of these conserved domains was also confirmed through UniProtKB.

### 3.4. Phylogenetic Analysis

**Figure 4:**
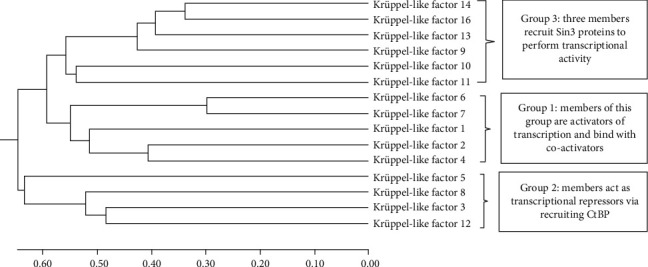
Phylogenetic analysis of KLF proteins by MEGA7. The evolutionary history was predicted by using the hierarchical clustering method unweighted pair group method with arithmetic mean (UPGMA). The evolutionary tree is calibrated to scale with the sum branch length of 7.54632578. In the optimal tree, the branch lengths are of the same unit that are used for evolutionary distances and computed by Poisson correction method by considering the number of amino acid substitutions per site. A total of 602 positions were present in the final amino acid sequences after removing ambiguous positions (pairwise deletion), and final evolutionary analyses indicating relationship of taxa were conducted in MEGA X [33].

The results of evolutionary conservation between KLF members in the form of phylogeny had placed KLF3 in group 2 based on its transcriptional activity and domain difference from other members. This division among members was confirmed by Molecular Evolutionary Genetics Analysis version 7.0 (MEGA7) software. The software relates members according to evolution and organizes them into three groups in the phylogenetic tree (see Figure 4).

### 3.5. 3-Dimensional Structure and Visualization of KLF3 Protein

**Figure 5:**
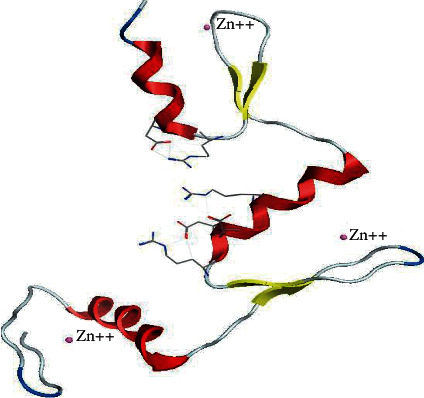
3D structure of KLF3. Alpha helices (red), beta sheets (yellow), coils (silver), and three zinc ions (depicted by a small circle) are connected via CH bonding with functional amino acids. However, no loops are shown in the protein. Model results from the Swiss Model server have depicted 80.72% sequence identity with relevance to KLF4.

**Figure 6:**
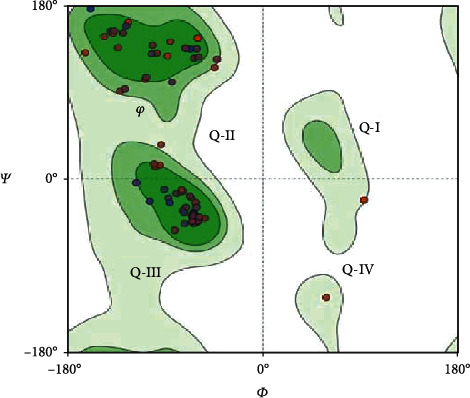
Ramachandran plot with -180 to +180 axes for determining secondary structures. The disfavored and allowed values of dihedral angles (*ψ* and *φ*) are shown in a two-dimensional plot. In quadrant I (Q-I), some left-handed helices are allowed (KLF3 lacks loops), quadrant II (Q-II) contained amino acids that favor beta strand conformation, quadrant III (Q-III) favors the region where right-handed alpha helices lie, and quadrant IV (Q-IV) has two amino acids (THR and ASP) clashing with the protein structure. Overall, this plot showed 724 interactions (bonds) within residues and 970 angles with 95.06% structure favorability.

**Figure 7:**
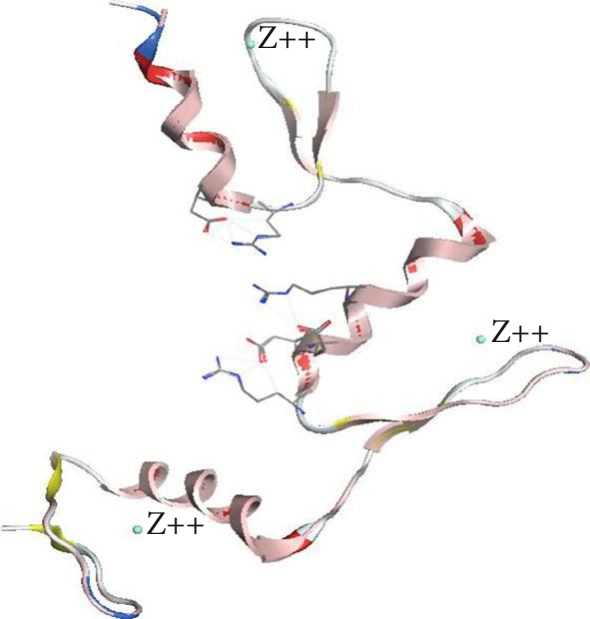
Superimposed structure of KLF3 (pink) and KLF4 as a template (red). The KLF3 chain shows matching with alpha helices of the template protein chain, but some residues deviate little from forming beta strands, so having a *Q*-score of 0.976. The model was visualized by UCSF Chimera 3.1 and depicted 80.72% identity between the target and template by superimposition.

The homology modeling between KLF3 and template KLF4 by using the Swiss Model server revealed 80.72% structural conservation. Along with sequence identity, the server also takes solvation factor, torsion angle, and structure quality estimate of KLF3 protein. The 3D structure of KLF3 is given in Figure 5. The quality estimate of the KLF3 model was observed by a Ramachandran plot and MolProbity scores which show that KLF3 protein structure is 95.06% favored by a Ramachandran plot; i.e., the predicted model was appropriate (see Figure 6). The built model of KLF3 was visualized in UCSF Chimera 1.13 for finding structural similarity between KLF4 (template) and the model (KLF3). The comparison of structure was evaluated by superimposition of the model (red) on the template (blue) as shown in Figure 7. The result of superimposition revealed that there exist 80.72% homology between both proteins after matching.

### 3.6. Expression of TPD52, KLF3, miR-124, and PKC*ε* in Blood of Breast Cancer Patients

**Figure 8:**
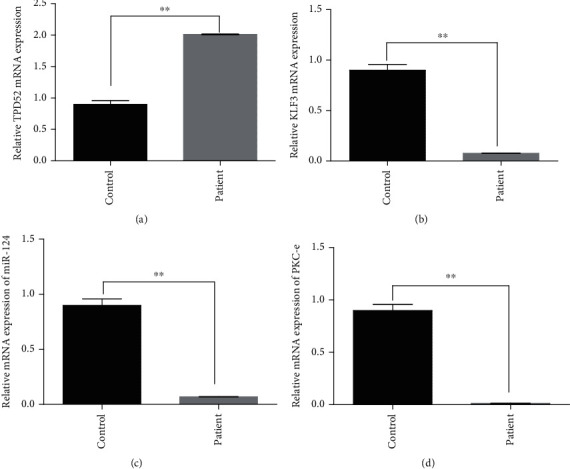
qPCR analysis of TPD52, KLF3, miR-124, and PKC*ε* expression in human breast cancer. Difference in expression relative to control: (a) high expression of TPD52; (b) low expression of KLF3; (c) decreased expression of miR-124; (d) reduced expression of PKC*ε*. The data expressed as fold change represents the mean ± standard error experiments performed in triplicate. Statistical significance was calculated by two-way ANOVA (^∗∗^*p* < 0.001).

Expression level of TPD52 was found to be elevated while KLF3, miR-124, and PKC*ε* genes were downregulated in breast cancer patients in comparison to controls. By using two-way analysis of variance (ANOVA), statistical significance was computed between both groups. The results indicated high expression of TPD52 (*p* = 0.0017) (Figure 8(a)) and low expression of KLF3 (*p* = 0.0048) (Figure 8(b)), miR-124 (*p* = 0.0048) (Figure 8(c)), and PKC*ε* (*p* = 0.0043) (Figure 8(d)) in blood of breast cancer patients as compared to healthy controls. In general, there was 2-fold increased expression of TPD52, and 0.076443-, 0.06969-, and 0.011597-fold decrease in the expression of KLF3, miR-124, and PKC*ε*, respectively, was observed in patients in comparison with healthy controls.

### 3.7. Relative Expression of TPD52, KLF3, miR-124, and PKC*ε* in Clinical-Pathological Features of Breast Cancer

**Figure 9:**
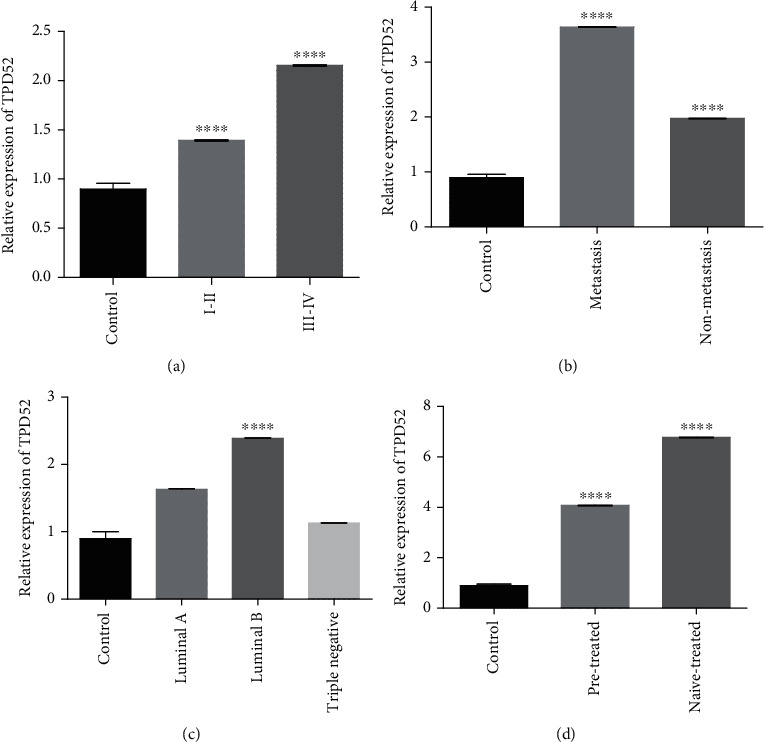
qPCR analysis of TPD52 expression in clinical characteristics of breast cancer cases. Correlation of TPD52 expression with (a) tumor stage, (b) metastasis, (c) receptor status, and (d) treatment condition. There was upregulated expression of TPD52 in lower tumor stages (I and II), nonmetastasis, cancer subtype (luminal B), and naïve-treated groups. The data expressed as fold change represents the mean ± standard error experiments performed in triplicate. Ordinary one-way ANOVA was used to establish significance (^∗∗∗∗^*p* < 0.0001).

The expression of TPD52, KLF3, miR-124, and PKC*ε* in breast cancer patients was measured (see Supplementary Table 1). The significant results of expression were found between tumor grades I/II and III/IV, metastatic vs. nonmetastatic features, pretreated vs. nontreated patients, and breast cancer subtypes (luminal A, luminal B, and triple negative). In the low-grade cancer group (grades I and II), the expression of TPD52 was found to be significantly elevated (*p* < 0.0001) as compared to that in the advance stage (III and IV). Moreover, the expression of TPD52 was significantly upregulated in nonmetastatic and metastatic groups (*p* < 0.0001). Among ER/PR/Her2-neu receptors, the most enhanced expression of TPD52 was present in the luminal B subtype group and the outcome was statistically significant (*p* < 0.0001). The patients that were naïve to any treatment (chemotherapy/radiation) carried advance expression (*p* < 0.0001) as compared to controls (see Figure 9).

**Figure 10:**
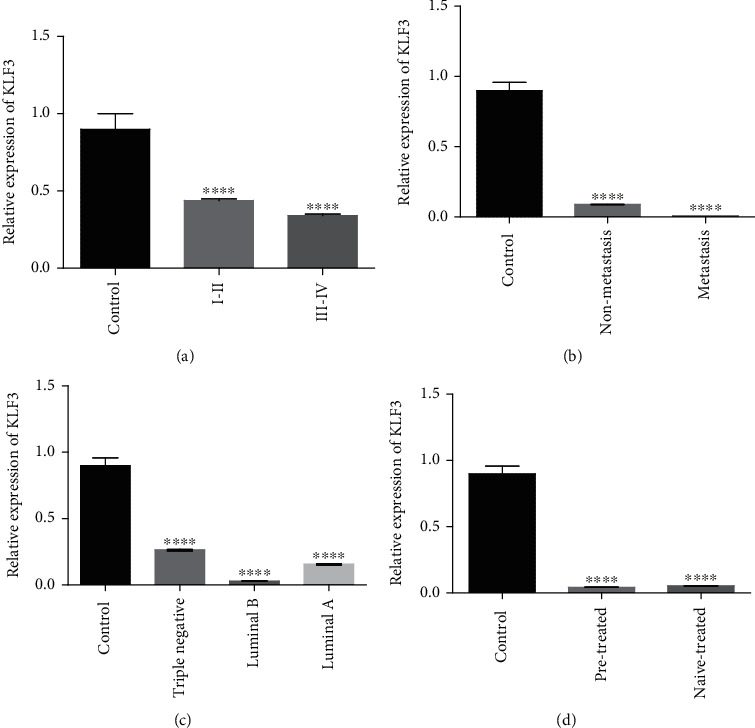
qPCR analysis of KLF3 expression with clinical characteristics of breast cancer cases. Correlation of KLF3 expression with (a) tumor stage, (b) metastasis, (c) receptor status, and (d) treatment condition. There is upregulated expression of KLF3 in lower tumor grades (I and II), nonmetastasis, cancer subtype (triple negative), and naïve-treated groups. The data expressed as fold change represents the mean ± standard error experiments performed in triplicate. Ordinary one-way ANOVA was used to establish significance (^∗∗∗∗^*p* < 0.0001).

**Figure 11:**
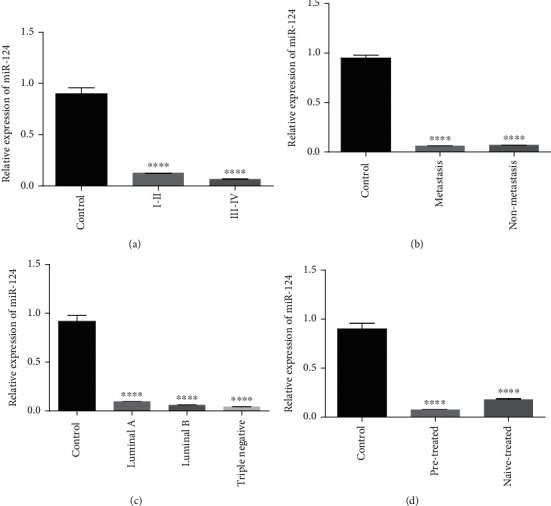
qPCR analysis of miR-124 expression with clinical characteristics of breast cancer cases. Correlation of miR-124 expression with (a) tumor grade, (b) metastasis, (c) receptor status, and (d) treatment condition. There is upregulated expression of miR-124 in low-grade tumor (I and II), nonmetastasis, cancer subtype (luminal A), and pretreated groups. The data expressed as fold change represents the mean ± standard error experiments performed in triplicate. Ordinary one-way ANOVA was used to establish significance (^∗∗∗∗^*p* < 0.0001).

As indicated in Figure 10, the expression of KLF3 was downregulated in stage I-IV, metastatic vs. nonmetastatic groups, in every molecular subtype (luminal A, luminal B, and triple negative), and in patients that were either on treatment or naïve to any kind of breast cancer treatment as compared to healthy controls. KLF3 expression was lower in the luminal B group in comparison to luminal A and triple negative groups, and its expression was further downregulated in the metastasis group in comparison to the nonmetastasis group. The expression of miR-124 was also downregulated in stage I-IV, metastatic vs. nonmetastatic groups, in every molecular subtype, and in patients that were either on treatment or naïve to any kind of breast cancer treatment as compared to healthy controls as shown in Figure 11. But compared to KLF3, the expression of miR-124 was downregulated in all molecular subtypes. miR-124 expression was also downregulated in both treated and nontreated groups in comparison to the control. But its expression was upregulated in the pretreated group relative to the nontreated group.

**Figure 12:**
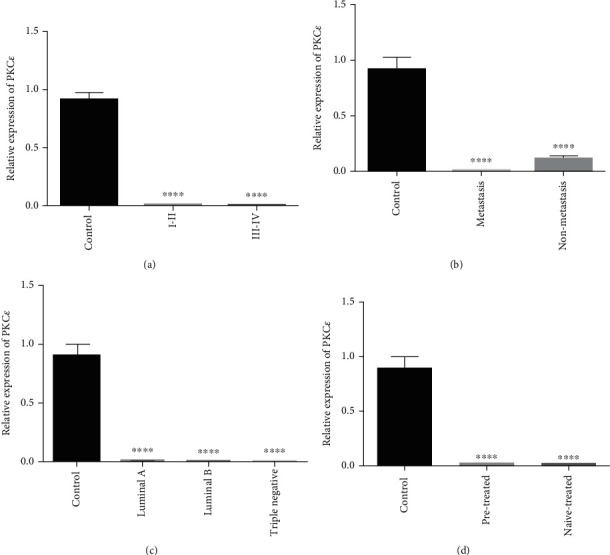
qPCR analysis of PKC*ε* expression with clinical characteristics of breast cancer cases. Correlation of PKC*ε* expression with (a) tumor stage, (b) metastasis, (c) receptor status, and (d) treatment condition. There is upregulated expression of PKC*ε* in high-grade tumor (III and IV), nonmetastasis, cancer subtype (luminal A), and naïve-treated groups. The data expressed as fold change represents the mean ± standard error experiments performed in triplicate. Ordinary one-way ANOVA was used to establish significance (^∗∗∗∗^*p* < 0.0001).

The expression of PKC*ε* was downregulated in all cancer stages, in molecular subtypes, in metastatic and nonmetastatic groups, and in patients that were either on treatment or naïve to any kind of breast cancer treatment as compared to healthy controls (see Figure 12).

### 3.8. Risk Factors for Breast Cancer

**Figure 13:**
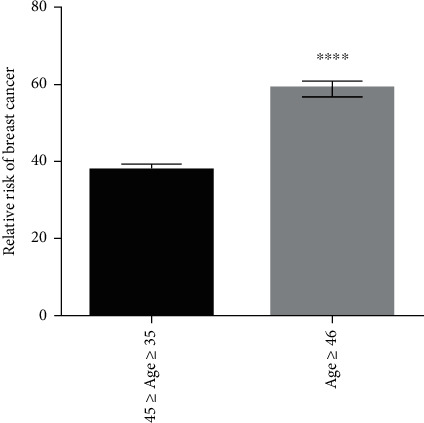
Risk of breast cancer relative to age. Mean age of patient with breast cancer. Risk is more in cases having age more than 46. Results are statistically significant with ^∗∗∗∗^*p* < 0.0001.

**Figure 14:**
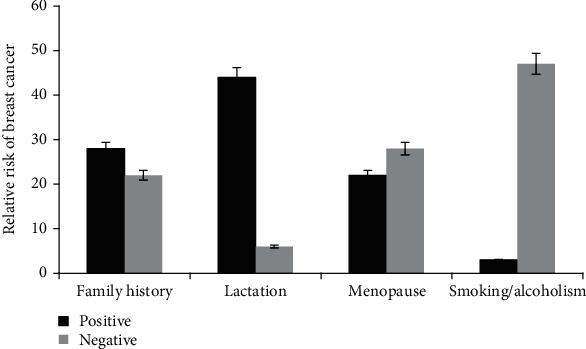
Study population of breast cancer exhibiting relative risks. Positive family history: *N* = 28. No/negative family history: *N* = 22. Positive lactation: *N* = 44. No lactation: *N* = 6. Females that were on menopause at the time of encountering breast cancer: *N* = 22. Nonsmokers/nonalcoholic: *N* = 47 among breast cancer cases.

Chance of breast cancer risk relative to age is shown in Figure 13. Mean age data shows that risk of breast cancer is higher in patients of age ≥ 46. The data is significant with *p* < 0.0001 calculated by Student's *t*-test. Simple data analysis regarding other risk factors (see Table 2) has shown that 44% patients with age more than 50 years were on postmenopause and 56% patients with age less than 49 were on premenopause at the time of encountering breast cancer. Six percent of the cases were those who consume cigarette while 94% patients had never took cigarette in their entire life. Moreover, 56% breast cancer patients had history of cancer in their family and 88% patients had breastfed their children (see Figure 14).

## 4. Discussion

Current diagnostic analysis of breast cancer includes pathological assessments through clinical examination as well as imaging techniques. Numerous techniques are being tried for diagnostic imaging and screening. Among them, some techniques are not in operational use and the rest often times fail to detect breast carcinoma at the treatable stage. Increasing cancer incidences and inadequacy of efficient diagnostic strategy have given rise to a strong need to establish novel diagnostic and prognostic approaches for breast cancer [34]. Hence, the present study intends to find the expression pattern of KLF3, miR-124, TPD52, and PKC*ε* in breast cancer patients which may serve as effective diagnostic biomarkers. Although the expression pattern of understudied genes have been analyzed individually and reported in several cancers following different signals, mechanisms, and factors, the coexpression of these genes in breast cancer was never reported. Further, results of the literature review and several other databases including the Protein Data Bank (RCSB PDB) revealed that the X-ray crystallography structure of KLF3 is yet to be determined. Hence, this study is conducted to establish 3D structure of KLF3 as well as the other features of KLF3 protein such as localization, conserved domains, and functional and phylogenetic analysis to build evolutionary relationship with other members of the family.

In silico results of our study have shown that KLF3 is located in the nucleus which was confirmed through UniProtKB (entry no. P57682). Evolutionary conservation was confirmed by the presence of conserved domains: three zinc fingers among members of KLFs, and it was also relatable to literature [18] and the UniProtKB database. Our study is also analogous to the Pearson review about the placement of KLF3 in group 2, and this study established the relation among family members in the form of a phylogenetic tree. The Ramachandran plot was used to assess the quality of 3D structure of KLF3, and the score of 95.06% depicted good quality of model. The proposed structure of KLF3 is 80.72% identical to the template model of KLF4. KLF4 anticancer activity in breast cancer is previously reported by several studies. It has a growth inhibitory role in ER^+^ breast cancer [35]. In vitro knockdown studies reported its role as a repressor of cell proliferation and angiogenesis [36, 37]. Considering the homology ratio of KLF3 with KLF4 and KLF4 tumor-suppressor role, it can be assumed that KLF3 might also function to inhibit carcinogenicity in breast cancer. KLF3 expression analysis revealed that its expression is downregulated with a fold change of 0.07. Present study expression analysis also suggests the tumor-suppressor role of KLF3 in breast cancer. Similar to our findings, significant reduction in expression of KLF3 is also reported in human metastatic sarcomas [19]. However, validation of KLF3 expression on larger cohort size and unraveling of its molecular role in breast cancer will further strengthen its significance as a biological marker.

Contrary to KLF3, *in vitro* results of the present study have shown the upregulated expression of TPD52 (2-fold) in breast cancer patients as compared to healthy controls. Our results were consistent with previous studies in which TPD52 expression was found to be significantly increased in ovarian and prostate cancer [38]. Moreover, the current study has depicted downregulation of miR-123 expression with a fold change of 0.06. Wang et al. [39] reported underexpression of miR-124 in pancreatic duct adenocarcinoma (PDAC) and showed its significant association with poor survival of patients. Their study outcomes further revealed that miR-124 targets the 3′-untranslated region of Rac1 (supposed tumor promoter in PDAC) directly. Rac1 miR-124-mediated downregulation causes inactivation of the MKK4-JNK-c-Jun signaling pathway and prevents uncontrolled cell proliferation. miR-124 serves as a tumor suppressor, and its silencing promotes cell proliferation in pancreatic cancer.

Various studies reported upregulation of PKC*ε* expression in numerous carcinomas such as prostate, brain, and lung cancer [40]. According to previous evidences on the expression of PKC*ε*, there exists an overexpression of PKC*ε* in breast cancer [41]. However, our study outcomes revealed a significant downregulation (0.01-fold) of PKC*ε* in breast cancer patients which are contradictory to past studies.

**Figure 15:**
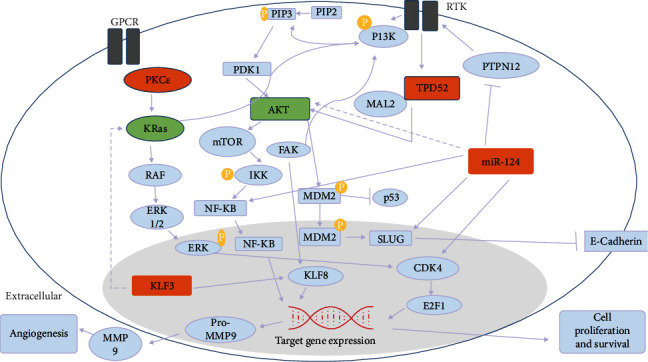
The involvement of the Akt and Kras pathway in breast cancer. Akt and Kras pathways were majorly involved in the progression of breast cancer. These two pathways were regulated by TPD52, miR-124, KLF3, PKC*ε*, and several other downstream effectors. The deregulated expression of TPD52, KLF3, miR-124, and PKC*ε* led to the overexpression of these two oncogenic pathways which in turn promoted cell survival and proliferation. Moreover, the partner genes of KLF3, TPD52, miR-124, and PKC*ε* were extracted through STRING software [53].

All of the genes involved in our study are known to be part of major cancer signaling pathways. Studies revealed that RTKs and GPCR are chiefly involved in modulating diverse signaling cascades. The downstream effector molecules of both receptors PI3K and Kras ultimately result in the activation of Akt and its associated pathways [9]. Akt signaling is a key cellular pathway responsible for cancer development, growth, survival, stage progression, drug resistance, and invasiveness [42]. RTKs are activated in the signaling pathways, so its activation leads to the stimulation of TPD52, located on chromosome 8. When TPD52 increases its copy number on one chromosome, it results in the increment of the MAL2 gene copy number [43]. These genes ultimately induce the activation of the Akt gene. Akt further activates downstream genes and leads the cell to its survival. miR-124—a tumor suppressor miRNA—plays a role in cell apoptosis, but in unfavorable conditions, the deregulated expression of miR-124 results in the proliferation of cells by aberrant activation of Kras and Akt pathways through the negative regulation of PTPN12 [13]. miR-124 downregulation results in the activation of cell cycle kinase (CDK4) which was an effector of Kras signaling and was activated by PKC*ε* as a result of GPCR. The activation of CDK4 signals the activation of the transcription factor E2F1 which in turn is responsible for cell proliferation [44]. In the same way, miR-124 also results in the increased expression of SLUG which is then bound to the promoter of E-cadherin and promotes cell invasion [45]. miR-124 activates the alpha catalytic subunit of PI3K, and the activated PI3K further promotes Akt activation [46]. Moreover, the deregulated expression of the transcriptional corepressor KLF3 is involved in Kras signaling [10]. Reduced expression of KLF3 in breast cancer upregulates the KLF8 gene, which is actually downstream of FAK. These sets of genes stimulate the expression of metalloproteinase 9 (MMP 9) [47] which further promotes angiogenesis and is the one source of nutrition for tumor cells (see Figure 15).

These molecules TPD52, KLF3, miR-124, and PKC*ε* have functional significance in the regulation of the Kras and Akt pathway. So, in the current study, the association between the expressions of TPD52, KLF3, miR-124, and PKC*ε* and clinicopathological attributes of breast cancer patients is also inferred. We found that the expression of TPD52 is higher in advanced-stage and metastatic breast cancer while the expression of KLF3 and miR-124 is lower in advanced clinical stages of tumor in comparison to initial stages. This suggests that the lower expression of KLF3 and miR-124 and enhanced expression of TPD52 have association with tumor metastatic potential and stage progression. Previously, elevated TPD52 expression in prostate cancer was reported to have association with tumor progression and metastasis [48]. Similarly, lower KLF3 and miR-124 expression was indicated in aggressiveness and metastatic uterine and cervical and ovarian cancers [49, 50]. Independent expression of TPD52 was evaluated by Roslan and colleagues in hormone receptor-positive breast cancer [51]. Their study findings also reported upregulated expression of TPD52 which is in accord with the present study result. But none of these studies evaluated the combined expression of these molecules in breast cancer, and the current study provides insight into functional relationship of these molecules with tumor aggressiveness and stage progression.

Similarly, we also investigated the expression pattern of these genes in treated and treatment-naïve patients. We found an elevated expression of TPD52 in treatment-naïve patients who were either on radiation therapy, chemotherapy, or chemoradiation therapy. This can be presumed that radio-, chemo-, or combined therapy in breast cancer influences the expression of oncogene TPD52 which suggests that TPD52 can be a potential therapeutic target for the metastatic and luminal B group of breast cancers.

The expression of PKC*ε* in the present study is found significantly downregulated in all tumor stages (I-IV), metastatic/nonmetastatic group of patients, molecular subtypes, and both naïve/treated groups. This data is in contrast to evidences provided by Pan and colleagues who reported elevated expression of PKC*ε* in breast cancer [41]. So, our study has reported a new response of the PKC*ε* gene in terms of its expression pattern.

The extent of genetics influence on the expression of oncogenes is boosted by a favorable environment. So, in this study, some of the risk factors that could possibly cause breast cancer were also analyzed. According to the analysis generated by our study, older age (age ≥ 46) is one of the factors that increase the likelihood of breast cancer. The facts and figures of 2017-2018 by the American Cancer Society (ACS) have also highlighted older age as a risk factor for breast cancer. According to previous studies, 74% females who were at their premenopausal age (35-49 years), before encountering breast cancer, were at definite risk of developing breast cancer [52].

The analysis of these genes provides novel insight into the coexpression pattern of these genes in different types and stages of breast cancer. Through extensive molecular studies, their role in disease progression can be understood. Further, the study outcomes also highlighted the potential application of these genes as biomarkers for cancer diagnosis and prognosis. In terms of therapeutic mediation in breast cancer, PI3K and Kras pathways are the very attractive targets because the components of these pathways are well suited for pharmacological intervention.

## 5. Conclusions

The RNA samples isolated from the blood of breast cancer patients were analyzed by qPCR for the expression of TPD52, KLF3, miR-124, and PKC*ε*. According to our results, the dysregulated expression pattern of all these genes is significantly coupled with disease progression. The fold change observed in samples has spotted the upregulation of TPD52 and downregulation of KLF3, miR-124, and PKC*ε* in breast cancer. Likewise, enhanced TPD52 expression and lower KLF3 expression in the metastatic group of patients in comparison to the control and nonmetastatic group can be considerably utilized for diagnosis purpose of breast cancer at the molecular level. As these genes might have application as a predictor of the stage, metastasis, receptor, and treatment status, they can be used as a new set of biomarkers for breast cancer diagnosis. Further, the in vitro knockdown studies and transcriptome analysis of these genes will provide deep insight into the functioning of these molecules in breast cancer.

## Figures and Tables

**(a):** 

Age (*n* = 50)	**30-39**	**40-49**	**50-59**	**60-69**	**>70**
	12	15	10	9	4

**(b):** 

Receptor status (ER/PR/Her2-neu)	**Luminal A**	**Luminal B**	**Triple negative**
	21	19	10

**(c):** 

Cancer stage	**I**	**II**	**III**	**IV**
	9	23	14	4
Cancer type	**IDC**	**DCIS**	**LCIS**	**IBC**
	28	5	4	1

**(d):** 

Treatment status	**Pretreated**	**Nontreated**
	39	11

IDC: invasive ductal carcinoma; DCIS: ductal carcinoma in situ; LCIS: lobular carcinoma in situ; IBC: inflammatory breast cancer.

**Table 2:** Distribution of breast cancer patients on the basis of risk factors.

Risk factors	Distribution of patients		
Age (*n* = 50) (*N* %)	**A** **g** **e** ≥ 46		45 ≥ **age** ≥ 35
	29 (58)		21 (42)
Family history (*N* %)	**Positive**		**Negative**
	28 (56)		22 (44)
	Siblings	5	
	Mother	8	
	Father	4	
	Maternal	4	
	Paternal	7	
Lactation (*N* %)	44 (88)		6 (12)
Menopause (*N* %)	22 (44) Age ≥ 50		28 (56) 49 ≥ age ≥ 35
Alcohol/smoking etc. (*N* %)	3 (6)		47 (94)

## Data Availability

The underlying raw data used to support the findings of this study are available from the corresponding author upon request.

## Supplementary Materials

Supplementary Materials Expression of TPD52, KLF3, miR-124, and PKC*ε* in different clinical attributes (such as cancer stage, type, and treatment status) of breast cancer is available in Supplementary Table 1.
