# Supplementary material for: Unravelling Structure, Localization, and Genetic Crosstalk of KLF3 in Human Breast Cancer
Source: Biomed Res Int. 2020 Dec 28;2020:1354381. doi: 10.1155/2020/1354381 (PMC7803292; doi:10.1155/2020/1354381)
Supplement: Supplementary Materials — Expression of TPD52, KLF3, miR-124, and PKCε in different clinical attributes (such as cancer stage, type, and treatment status) of breast cancer is available in Supplementary Table 1. [file 1354381.f1.docx]

Supplementary Table 1: Expression of TPD52, KLF3, miR-124 and PKC-e in Clinical Attributes of Breast Cancer.

| **Clinico-pathological Features** | **TPD52 Expression Fold Change with p<0.0001** | **KLF3 Expression Fold Change with p<0.0001** | **miR-124 Expression Fold Change with p<0.0001** | **PKC-e Expression Fold Change with p<0.0001** |
| --- | --- | --- | --- | --- |
| **Stage 1-2** | 2.1549 | 0.4380 | 0.118383 | 0.00819 |
| **Stage 3-4** | 1.3934 | 0.3495 | 0.066401 | 0.008818 |
| **Metastatic**  **Non-Metastatic** | 3.6378 | 0.008038 | 0.062597 | 0.004695 |
|  | 1.9679 | 0.089762 | 0.070342 | 0.113362 |
| **Luminal A**  **Luminal B**  **Triple negative** | 1.6218 | 0.156357 | 0.096896 | 0.011656 |
|  | 2.3869 | 0.029477 | 0.062567 | 0.004664 |
|  | 1.1299 | 0.264007 | 0.044941 | 0.007216 |
| **Pre-Treated**  **Naïve-Treated** | 4.0764 | 0.042077 | 0.075977 | 0.009585 |
|  | 6.7596 | 0.054242 | 0.026237 | 0.007114 |
